# Assessment of Harms Reporting Quality in Randomized Controlled Trials of Aesthetic Rhinoplasty: A Systematic Review

**DOI:** 10.1093/asjof/ojae057

**Published:** 2024-07-22

**Authors:** Tara Behroozian, Victor Ripan, Patrick Kim, Morgan Yuan, Lucas Gallo, Kathryn Ulhman, Mark McRae, Dale Podolsky, Jamil Ahmad

## Abstract

**Background:**

Rhinoplasty is one of the most common aesthetic plastic surgery procedures. Complications can lead to both aesthetic and functional impairments. The Consolidated Standards of Reporting Trials (CONSORT) Harms statement was developed to promote improved reporting of harm across randomized controlled trials (RCTs).

**Objectives:**

The aim of this systematic review is to assess harms reporting quality across RCTs on aesthetic rhinoplasty.

**Methods:**

A literature search was conducted in Ovid MEDLINE and Embase databases (January 1, 2005 to August 4, 2023). RCTs which compared 2 or more interventions in rhinoplasty with primarily aesthetic indications and assessed patient-important outcomes were included. The reporting quality was assessed by following a 40-item checklist endorsed by the 2022 CONSORT Harms Extension update.

**Results:**

A total of 58 RCTs met the inclusion criteria. Fifteen RCTs addressed harms of treatment in some capacity. Overall, the reporting quality across RCTs was poor, with a median CONSORT Harms score of 33% (range, 16%-83%). A reporting adherence of ≥50% was met by only 8 studies. There was no significant difference in reporting adherence between studies based on journal endorsement of CONSORT or industry vs nonindustry funding sources (*P* > .05). A high journal impact factor was significantly associated with a higher reporting quality (*P* = .044).

**Conclusions:**

CONSORT Harms reporting adherence was poor across the majority of included RCTs. Future trials on aesthetic rhinoplasty should aim to follow the reporting recommendations endorsed by the CONSORT Harms statement to increase transparency and minimize heterogeneity in harms reporting across studies.

**Level of Evidence: 1:**

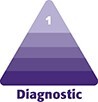

Rhinoplasty is one of the most common aesthetic surgery procedures performed by plastic surgeons, and demand for the procedure has been steadily increasing over recent decades.^1^ This demand necessitates ongoing development of new techniques and patient safety strategies to improve aesthetic and functional outcomes which should ideally be evaluated using randomized controlled trials (RCTs).^2,3^ Despite the positive aesthetic impact of plastic surgery on patients, there are several potentially serious complications associated with rhinoplasty, including epistaxis, infection, deformity, septal hematoma, skin necrosis, graft rejection, and intracranial injury.^1^

The field of plastic surgery is often criticized for a lack of evidence-based clinical decision-making,^4^ and calls have been made to improve reporting quality of RCTs in plastic surgery.^5^ In comparison with other fields, high-quality RCTs in plastic surgery are conducted less frequently, with only 2% of articles in plastic surgery journals consisting of RCTs.^6^ In aesthetic surgery, there is often a lack of clinical equipoise and difficulty establishing a placebo given the visible nature of the condition being treated.^6^ Additional factors, such as learning curves and high costs, contribute to challenges in conducting plastic surgery trials, leading to a lack of RCTs in aesthetic surgery and relying on anecdotal experience rather than quantitative evidence.^6,7^ As such, there is a need for greater transparency in this area. In fact, one of the preconditions for conducting high-quality research in plastic surgery is to report study findings according to the appropriate reporting guidelines.^8^ The need for consistent reporting is especially important in aesthetic trials, which are often industry funded and may suffer from biases in reporting as a result.^9^

Despite the utility of RCTs in guiding clinical practice, reporting of harms and adverse events in RCTs remains inconsistent across disciplines.^10-14^ Without accurate and consistent reporting of RCT findings and the harms of interventions, both clinicians and patients are unable to provide fully informed decisions regarding treatment. To enhance transparency in reporting while minimizing systematic errors across RCTs, the Consolidated Standards of Reporting Trials (CONSORT) group first introduced a standardized checklist in 1996, which has since been most recently updated in 2010.^15^ In 2004, the CONSORT Harms extension checklist was published to promote improved reporting of harms across RCTs, which has since been updated in 2022.^16,17^ The CONSORT Harms 2022 statement integrates harm-focused items into the preexisting CONSORT statement, whereby a harm is defined as any unintended event which may impact patients’ outcomes, quality of life, and overall experience with treatment.^16^

To our knowledge, there have been no previous studies examining the reporting quality and harms reporting of aesthetic rhinoplasty RCTs. Given the importance of harms in clinical decision-making on aesthetic interventions, this systematic review aims to assess the compliance of RCTs on aesthetic rhinoplasty to the CONSORT Harms 2022 statement. We additionally sought to investigate whether adherence to the CONSORT Harms was influenced by journal endorsement of CONSORT, trial funding source, and journal impact factor.

## METHODS

This systematic review is part of a larger review aiming to assess the reporting quality of RCTs in all aesthetic interventions; the review protocol has been outlined and registered in the PROSPERO review registry (CRD42023454002). It was conducted in accordance with the Preferred Reporting Items for Systematic Reviews and Meta-Analyses (PRISMA) statement (Appendix A).^18^

### Search Strategy and Selection Criteria

Ovid MEDLINE and Embase databases were searched from January 1, 2005 to August 4, 2023 using subject headings and keywords to capture RCTs investigating aesthetic interventions, such as “esthetics,” “randomized controlled trial,” “esthetic surgery,” and the MeSH descriptor “cosmetic techniques.” The year 2005 was chosen as the original CONSORT Harms extension checklist was published in November 2004.^17^ The detailed search strategy has been included in Appendix B. Gray literature was also screened for additional studies to be included in the review. The literature search was completed by authors who have extensive experience working with medical librarians in developing search strategies.

All RCTs published in the English language were included if they (1) compared 2 or more surgical or nonsurgical interventions in rhinoplasty with primarily aesthetic indications and (2) reported patient-important outcomes. Patient-important outcomes were defined as any outcomes that are directly relevant to patients, such as safety, adverse events, symptoms, and quality of life.^8,19^ The following exclusion criteria were applied: (1) observational or nonrandomized interventional study designs, (2) conference abstracts, letters, secondary analyses, (3) nonhuman models, or (4) lifestyle interventions.

In accordance with the PRISMA,^20^ 2 reviewers independently screened through titles, abstracts, and full texts identified through the search strategy for inclusion in the analysis. A third author was consulted as needed to resolve any discrepancies.

### Data Extraction and Analysis

Following screening of titles, abstracts, and full texts, a piloted data extraction form was used, in which the first 20% of articles were extracted in duplicate and cross-referenced for discrepancies. The remaining articles were extracted independently and in duplicate.

Among included studies, the following study characteristics were collected: country, year, journal, CONSORT endorsement of journal, blinding, single- vs multicenter, sample size, intervention type, comparator, primary trial objective (efficacy vs harm), patient-important outcomes assessed, and funding source. Using the Journal Citation Reports database, journal impact factor was extracted for the year 2022 or the most recent available year if 2022 was unavailable. The CONSORT Harms 2022 checklist items were extracted for each study. The reporting of common expected postoperative effects of treatment, such as edema, ecchymoses, pain, and degree of nasal obstruction, were not considered as harms to remain in keeping with the CONSORT definition of harms as being unintended and as these temporary symptoms are considered an anticipated part of the normal postoperative course for all patients to some extent.^21^

For each RCT, a score of “1” (indicating “yes”), “0” (indicating “no”), or “NA” (indicating “not applicable”) was assigned for each item on the CONSORT Harms 2022 checklist. It was not suitable to assign a total score for each study given that not all items were applicable for each one, such as 3b (*changes to methods after trial commencement*), 6c (*how nonprespecified outcomes were identified*), and 7b (*interim analyses and stopping guidelines*). As such, a percentage was calculated based on the number of applicable items that were adequately addressed. Checklist items were all weighed as equally important, in accordance with previously published reviews that assessed CONSORT Harms 2022 adherence.^10,12,14^

The median number of checklist items that were adequately addressed in each RCT was calculated. Two-sample *T*-tests using unequal variances were used to assess whether there was a significant difference in reporting in trials that were industry funded vs nonindustry funded, trials published in journals with the 5 highest impact factors vs all other journals, and trials published in CONSORT-endorsing vs nonendorsing journals. The top journals based on the 5 highest impact factors included *Rhinology*, *JAMA Facial Plastic Surgery*, *Plastic and Reconstructive Surgery*, *Journal of Otolaryngology—Head and Neck Surgery*, *Aesthetic Surgery Journal*, and *Journal of Cranio-Maxillo-Facial Surgery*. All analyses were performed using Microsoft Excel. The risk of bias and certainty of evidence of included studies were not assessed as this review did not assess the individual study outcomes.

## RESULTS

### Literature Search Results

**Figure 1.**
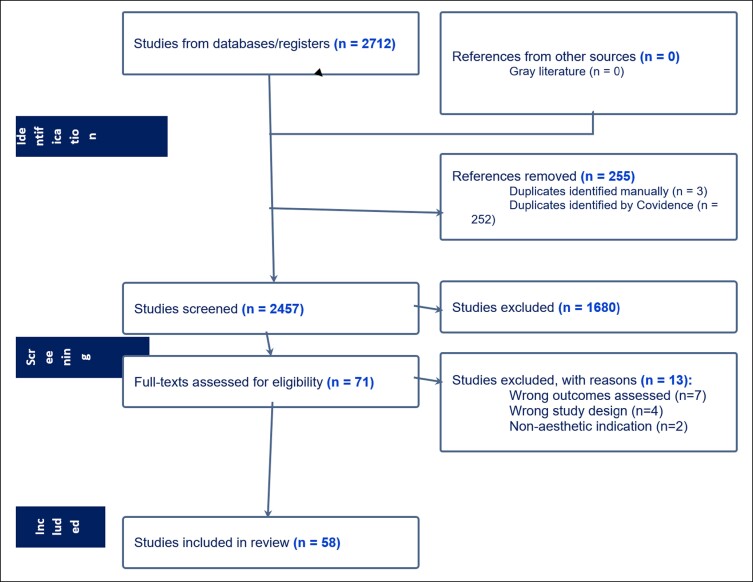
Literature search results.

As part of a larger review on all types of aesthetic and cosmetic interventions, a total of 2712 articles were identified in the initial database search, with 2457 remaining after duplicates were removed. After screening by titles, abstracts, and full-text articles, a total of 58 were identified for inclusion. PRISMA flow diagram is available in Figure 1.

Characteristics of included RCTs are summarized in Table 1, and details about the individual trials are summarized in Appendices C and D. The majority of RCTs were completed in the Middle East (Iran, Turkey, Egypt; *n* = 38, 66%), Europe (Italy, Portugal, Switzerland; *n* = 9, 16%), and North America (USA, Canada; *n* = 6, 10%). A total of 3996 patients were included across studies. Thirty-two studies (55%) were published in CONSORT-endorsing journals, whereas 26 (45%) were published in non-CONSORT-endorsing journals. Most RCTs included an efficacy outcome as the primary trial objective (*n* = 56, 97%), with only 2 focusing primarily on harms. Funding sources included industry (*n* = 3, 5%), university (*n* = 3, 5%), government (*n* = 2, 3%), and none (*n* = 24, 45%) among studies that reported this information.

**Table 1.** Characteristics of Included Studies

Demographics	No. of trials (%)
Total sample size	
Mean	68.9
Range	10-250
Year of publication	
2005-2009	5 (8.6)
2010-2014	4 (6.8)
2015-2019	26 (44.8)
2020-Present	27 (46.6)
Country of author	
Middle East	38 (65.5)
Europe	9 (15.5)
North America	6 (10.3)
East Asia	3 (5.2)
South Asia	1 (1.7)
South America	1 (1.7)
Blinding	
Single	27 (46.5)
Double	13 (22.4)
Nonblinded	18 (31.0)
Single- or multicenter	
Single center	57 (98.3)
Multicenter	1 (1.7)
Primary trial objective	
Efficacy	52 (90.0)
Efficacy and harm	4 (6.7)
Harm	2 (3.4)
Journal Impact Factor	
Mean	2.30
Range	0.5-6.7
Journals	
*Aesthetic Plastic Surgery*	10 (17.2)
*Journal of Craniofacial Surgery*	9 (15.5)
*Journal of Oral and Maxillofacial Surgery*	5 (8.6)
Others	34 (58.6)
CONSORT endorsement of journals	
Only CONSORT endorsed	4 (6.9)
CONSORT and extensions endorsed	11 (19.0)
Reporting guidelines through EQUATOR network endorsed	17 (29.3)
No endorsement	26 (44.8)
Funding source	
Government	2 (3.4)
University	3 (5.2)
Industry	3 (5.2)
None	24 (41.4)
Not reported	26 (44.8)

CONSORT, Consolidated Standards of Reporting Trials; EQUATOR, Enhancing the QUAlity and Transparency Of health Research (EQUATOR) Network.

Common outcomes assessed included: edema (*n* = 24), ecchymoses (*n* = 22), patient satisfaction (*n* = 18), nasal patency/obstruction (*n* = 10), pain (*n* = 9), and aesthetic appearance (*n* = 7). Fifteen RCTs assessed adverse events or complications of treatment, which were considered to be harms, such as tissue necrosis, infections, graft rejection or extrusion, hematoma, and septal perforation. Among these, 10 outlined specific objectives for outcomes of both benefits and harms (CONSORT Item 2b). Twenty-one RCTs reported that zero harm events occurred (Item 17c), whereas 11 reported the occurrence of actual harm events.

### CONSORT Harms Adherence

The median calculated CONSORT Harms score was 33% (range, 16%-83%) across all RCTs, with only 2 RCTs achieving an “adequate” level of reporting. Adherence to each individual item is summarized in Table 2. The greatest reporting adherence was among Items 2a (*scientific background and explanation of rationale*; *n* = 58/58, 100%), 4a (*eligibility criteria for participants*; *n* = 58/58, 100%), 5 (*sufficient details about interventions*; *n* = 56/58, 97%), 11b (*similarity of interventions*; 34/34, 100%), and 22 (*interpretation consistent with results*; 58/58, 100%). The 6 items with the poorest adherence were the following: 17a2 (*rationale for omitted outcomes*; *n* = 0/58, 0%), 17b (*absolute and relative effect sizes*; *n* = 1/58, 1.72%), 17a (*estimated effect size and precision*; *n* = 2/58, 3.45%), 21 (*generalizability*; *n* = 2/58, 3.45%), 10 (*implementation of random allocation sequence and participant enrollment/assignment*; *n* = 4/58, 6.90%), and 24 (*access to trial protocol*; *n* = 4/58, 6.90%). Of 26 RCTs that failed to report any harms, none provided an alternative source to access harms data, such as supplementary files. In studies where Item 15 (*table with baseline demographics/clinical characteristics*) was adequately reported, minimal demographic data were provided, often solely including patients’ age and sex. Items 3b (*changes to methods after trial commencement*), 6c (*identification of nonprespecified outcomes*), 7b (*interim analyses and stopping guidelines*), and 14b (*why the trial ended or was stopped*) were not applicable in any of the studies.

**Table 2.** CONSORT Harms Reporting Adherence Across Included RCTs

CONSORT harms 2022 checklist item			No. of RCTs where item was reported adequately among applicable studies (%)	No. of RCTs where item was reported inadequately among applicable studies (%)
Title and abstract	1a	Identification as a randomized trial in the title	20/58 (34)	38/58 (66)
	1b	Structured summary of trial design, methods, results of outcomes of benefits and harms, and conclusions (for specific guidance see CONSORT for abstracts)	10/58 (17)	48/58 (83)
*Introduction*				
Background and objectives	2a	Scientific background and explanation of rationale	58/58 (100)^a^	0/58 (0)
	2b	Specific objectives or hypotheses for outcomes of benefits and harms	11/58 (19)	47/58 (81)
*Methods*				
Trial design	3a	Description of trial design (such as parallel, factorial) including allocation ratio	39/58 (67)	19/58 (33)
	3b	Important changes to methods after trial commencement (such as eligibility criteria), with reasons	NA	NA
Participants	4a	Eligibility criteria for participants	58/58 (100)^a^	0/58 (0)
	4b	Settings and locations where the data were collected	18/58 (31)	40/58 (69)
Interventions	5	The interventions for each group with sufficient details to allow replication, including how and when they were actually administered	56/58 (97)^a^	2/58 (3)
Outcomes	6a	Completely defined prespecified primary and secondary outcomes, for both benefits and harms, including how and when they were assessed	14/58 (24)	44/58 (76)
	6b	Any changes to trial outcomes after the trial commenced, with reasons	1/1 (100)^a^	0/1 (0)
	6c	Describe if and how nonprespecified outcomes of benefits and harms were identified, including any selection criteria, if applicable	NA	NA
Sample size	7a	How sample size was determined	12/58 (21)	46/58 (79)
	7b	When applicable, explanation of any interim analyses and stopping guidelines	NA	NA
Randomization: sequence generation	8a	Method used to generate the random allocation sequence	28/58 (48)	30/58 (52)
	8b	Type of randomization; details of any restriction (such as blocking and block size)	19/58 (33)	39/58 (67)
Allocation concealment mechanism	9	Mechanism used to implement the random allocation sequence (such as sequentially numbered containers), describing any steps taken to conceal the sequence until interventions were assigned	15/58 (26)	43/58 (74)
Implementation	10	Who generated the random allocation sequence, who enrolled participants, and who assigned participants to interventions	4/58 (7)	54/58 (93)
Blinding	11a	If done, who was blinded after assignment to interventions (eg, participants, care providers, those assessing outcomes of benefits and harms) and how	16/40 (40)	24/40 (60)
	11b	If relevant, description of the similarity of interventions	34/34 (100)^a^	0/34 (0)
Statistical methods	12a	Statistical methods used to compare groups for primary and secondary outcomes of both benefits and harms	9/58 (16)	49/58 (84)
	12b	Methods for additional analyses, such as subgroup analyses and adjusted analyses	1/1 (100)^a^	0/1 (0)
*Results*				
Participant flow (a diagram is strongly recommended)	13a	For each group, the numbers of participants who were randomly assigned, received intended treatment, and were analyzed for outcomes of benefits and harms	21/58 (36)	37/58 (64)
	13b	For each group, losses and exclusions after randomization, together with reasons	7/12 (58)	5/12 (42)
Recruitment	14a	Dates defining the periods of recruitment and follow-up for outcomes of benefits and harms	11/58 (19)	47/58 (81)
	14b	Why the trial ended or was stopped	NA	NA
Baseline data	15	A table showing baseline demographic and clinical characteristics for each group	25/58 (43)	33/58 (57)
Numbers analyzed	16	For each group, number of participants (denominator) included in each analysis of outcomes of benefits and harms and whether the analysis was by original assigned groups and if any exclusions were made	19/58 (33)	39/58 (67)
Outcomes and estimation	17a	For each primary and secondary outcome of benefits and harms, results for each group, and the estimated effect size and its precision (such as 95% CI)	2/58 (3)	56/58 (97)
	17a2	For outcomes omitted from the trial report (benefits and harms), provide rationale for not reporting and indicate where the data on omitted outcomes can be accessed	0/58 (0)	58/58 (100)
	17b	Presentation of both absolute and relative effect sizes is recommended, for outcomes of benefits and harms	1/58 (2)	57/58 (98)
	17c	Report zero events if no harms were observed	21/47 (45)	26/47 (55)
Ancillary analyses	18	Results of any other analyses performed for outcomes of benefits and harms, including subgroup analyses and adjusted analyses, distinguishing prespecified from exploratory	1/2 (50)	1/2 (50)
Harms	19	All important harms or unintended effects in each group (for specific guidance see CONSORT for harms)	18/58 (31)	40/58 (69)
*Discussion*				
Limitations	20	Trial limitations, addressing sources of potential bias related to the approach to collecting or reporting data on harms, imprecision, and, if relevant, multiplicity or selection of analyses	7/58 (12)	51/58 (88)
Generalizability	21	Generalizability (external validity, applicability) of the trial findings	2/58 (3)	56/58 (97)
Interpretation	22	Interpretation consistent with results, balancing benefits and harms, and considering other relevant evidence	58/58 (100)^a^	0/58 (0)
*Other information*				
Registration	23	Registration number and name of trial registry	7/58 (12)	51/58 (88)
Protocol	24	Where the full trial protocol and other relevant documents can be accessed, including additional data on harms	4/58 (7)	54/58 (93)
Funding	25	Sources of funding and other support (such as supply of drugs), role of funders	32/58 (55)	26/58 (45)

^a^CONSORT, Consolidated Standards of Reporting Trials. Item was reported adequately (>75% of studies where item was applicable).

Among 32 (55%) studies published in journals where CONSORT (either alone, with its extensions, or through the Enhancing the QUAlity and Transparency Of health Research [EQUATOR] Network) was endorsed, the median CONSORT Harms score was 37%. In the 26 (45%) studies published in journals with no CONSORT endorsement, the median CONSORT Harms score was 32%. There was no significant difference in adherence between studies based on CONSORT endorsement of the journal (CONSORT endorsing vs nonendorsing, *P* = .31) or funding source (industry vs nonindustry, *P* = .12; Table 3). CONSORT Harms adherence was significantly higher in the 5 journals with the highest impact factors (*P* = .044). As there were only 3 RCTs^22-24^ published since the publication of the 2022 update to the CONSORT Harms checklist, which was in April 2023,^16^ it was not possible to determine whether there was a significant difference in reporting practices before and after the 2022 update to the CONSORT Harms checklist.

**Table 3.** CONSORT Adherence According to Journal Impact Factor and Industry Funding Source

	Published in journals with the top 5 impact factors			Industry funded			Consort-endorsed journal		
	Yes (*n* = 13)	No (*n* = 45)	*P*-value	Yes (*n* = 3)	No (*n* = 55)	*P*-value	Yes (*n* = 32)	No (*n* = 26)	*P*-value
Mean CONSORT Harms Score	46%	33%	.044	65%	35%	.12	38%	34%	.31

A *P*-value < .05 is considered statistically significant.

## DISCUSSION

The present study highlights that RCTs investigating interventions for aesthetic rhinoplasty have a poor overall reporting adherence, with only 8 studies (14%) having an adherence to the CONSORT Harms statement of ≥50%. Unfortunately, the inadequate reporting quality among studies is a cause for concern as the lack of transparency surrounding the harms of an intervention makes it difficult for clinicians and patients to make informed treatment decisions. Given that aesthetic rhinoplasty procedures are not considered medically necessary treatments, it is especially important that patients be well informed of the potential risks associated with treatment.

RCTs are deemed a “gold standard” of evidence for comparing different interventions for treatment benefit. Unfortunately, there is a tendency for authors to over-emphasize the benefits of a given treatment while omitting the associated harms.^16^ The CONSORT Harms statement was developed with the intention of providing researchers with clear instructions on the type of information to report in their trials to minimize methodological flaws and enhance transparency surrounding harms. It was created first as an extension to the main CONSORT checklist as the main checklist includes only one item on harms.^16,17^ However, given the lack of adherence to the 2004 harms extension among published trials, it was updated in 2022 to integrate harm-focused items within the main checklist itself.^16^ As per the CONSORT group, it is recommended that the current 2022 CONSORT Harms statement be used to appraise reporting quality until the main checklist is updated to reflect the importance of harms reporting in RCTs.^16^

In the present review, the 2022 harms statement was followed in accordance with the CONSORT recommendations given that aesthetic rhinoplasty is a nonessential procedure with potential harms that can lead to both functional and aesthetic complications. A 2020 systematic review of adverse events of aesthetic rhinoplasty identified some of the most common harms reported in the literature, including numbness/paresthesia (event rate: 4.0%-49.1%), skin discoloration (1.7%-21.8%), nasal airway obstruction (0%-23.7%), bleeding (0%-23.4%), need for revision surgery (0%-10.9%), hypertrophic scarring (0.55%-9.1%), and seroma (7.4%).^25^ Although potential complications are rarely devastating, these have shown to be associated with postoperative decision regret among patients.^26,27^ As such, the transparent reporting of harms associated with aesthetic rhinoplasty is critical in ensuring that provider and patient decision-making is evidence-based and to minimize instances of decision regret.

In 2021, the Evidence-based Performance Measures for Rhinoplasty were published by the American Society of Plastic Surgeons, the American Academy of Otolaryngology–Head and Neck Surgery, and the American Academy of Facial Plastic and Reconstructive Surgery.^28^ Four performance measures were identified. Three performance measures were process measures: (1) motivations and expectations of the procedure, (2) airway assessment, and (3) nonnarcotic shared decision-making strategies for pain management. One of the performance measures was a process measure: (1) patient satisfaction. Outcome factors related to complications were not included because of their infrequent occurrence; this may be because of the true frequency of the complications or related to the potential for publication bias against reporting negative consequences.

The poor reporting quality among included RCTs can be attributed to a multitude of factors. First, most studies did not prespecify what harms they would assess and how these outcomes would be collected, and instead only reported that no harms occurred (Item 17c). The CONSORT Harms statement recommends against this nonsystematic way of collecting data, as it relies on self-reporting by participants and is associated with an inherent risk of bias.^16^ Next, in studies with a small-to-medium sample size, a lack of statistical power may have limited the ability for establishing significant or nonsignificant findings in harms reporting.^29^ Limits on article word counts and a lack of CONSORT endorsement by journals may have also contributed to inadequate harms reporting. The overall generalizability of studies may have additionally been limited because of the omission of patients with known risk factors for postoperative complications from some study populations, such as smokers or diabetics.

Notably, the majority of studies on aesthetic rhinoplasty were conducted in the Middle East (*n* = 38, 65.5%), which is not surprising as previous studies have highlighted the high demand for rhinoplasty in Middle Eastern countries.^30^ Research ethics requirements in Middle Eastern countries have reportedly been less rigid than those in Europe and North America, likely because of a lower adherence to international guidelines, such as the Declaration of Helsinki and the International Conference of Harmonization—Good Clinical Practice.^31^ The less stringent ethical guidelines in Middle Eastern countries may explain the poor reporting quality of the majority of the included studies. RCT reporting quality has been compared across geographical regions in other contexts, such as endodontics, in which RCTs originating from the Middle East, Asia, North America, and South America were more likely to have lower reporting quality than RCTs from Europe.^32^ Although diverse geographic representation should continue to be encouraged in research, it is important to acknowledge how institutional regulations may lead to differences in reporting quality.

Among items which were met by the majority of studies, some improvements could have been made with regards to reporting. Item 15 on baseline data was met by 43% (*n* = 25/58) of studies; however, the baseline demographic and clinical characteristics that were reported were minimal and mostly only included age and sex. Additional factors that could have been reported include any baseline risk factors for operative complications (eg, smoking status, bleed risk), history of nasal trauma, and nasal deformity type, among others. The inclusion of this baseline information would have given readers a better picture of the patient population and any patient-specific factors that may have contributed to unintended outcomes. Additionally, very few studies followed the CONSORT recommendation to include a flow diagram that illustrates the phases of each treatment group throughout the study (enrollment, intervention allocation, follow-up, and data analysis). This made it challenging to discern the total number of patients included in the final data analysis, especially with regards to harms outcomes. Most studies also failed to satisfy Item 21 on generalizability (*n* = 56/58, 97%), which was expected as the nature of the findings of interventions on aesthetic rhinoplasty is difficult to generalize to other areas of research. For Item 19 (*all important or unintended effects in each group*), studies were considered to have met the criteria if they reported one or more major harms given the lack of a core outcome set for harms reporting in rhinoplasty. It is important to note, however, that reporting of a single harm does not constitute all important harms, so the reporting adherence of Item 19 may be overrepresented.

Although no reviews have yet been published using the 2022 CONSORT Harms tool, 13 have assessed harms reporting adherence in RCTs using the 2004 version.^33^ Among these trials, adherence to the CONSORT Harms checklist remained below 50% among the majority of the items.^33^ The items with the poorest adherence among RCTs published after 2004 included Item 2 (*addressing harms and benefits in the introduction*; 35%), Item 5 (*description of plans for presenting and analyzing harms*; 31%), and Item 9 (*description of any subgroup or exploratory analyses*; 13%). The items from the 2004 checklist differ from the updated statement and therefore cannot be compared directly. However, there were evident similarities in the findings of our review. Among our included RCTs, harms objectives were seldom reported in the introduction, and there was often a lack of prespecified outcomes and outcome measurements when harms were assessed or reported.

Surgical and nonsurgical techniques for aesthetic rhinoplasty are constantly evolving, with several trials currently registered on ClinicalTrials.gov investigating various interventions. Future RCTs should aim to adhere to the CONSORT Harms statement by specifying harms that will be assessed a priori and reporting them in a systematic manner. It may also be beneficial to standardize a minimum number of outcomes, with an emphasis on harms outcomes, that should be assessed in all future trials on aesthetic rhinoplasty. Journals play an important role in improving reporting quality as well. They are encouraged to endorse adherence to the EQUATOR Network, which recommends the CONSORT Harms tool as well as other CONSORT extensions. The journals should not only endorse a higher standard of reporting, but should also make active efforts to ensure that authors are adhering to the reporting requirements as the present review highlighted that poor adherence was consistent across both CONSORT-endorsing and nonendorsing journals.

Although the CONSORT Harms tool is widely accepted and used in appraising the quality of RCTs, it is important to acknowledge its limitations. Despite efforts to clearly instruct users on appropriate use of the tool, the CONSORT items remain subjective in nature, which may have resulted in bias when assessing study reporting quality. Although reporting quality was poor among the majority of included studies, this does not mean that the studies were conducted poorly in actuality. Rather, their methodology and results were difficult to appraise from a reader's perspective. As such, the findings of these studies should not be entirely discredited. In addition to the subjective nature of reporting quality assessment, there are additional limitations specific to this review that should be addressed. By excluding non-English studies, we may have failed to capture all relevant RCTs on this topic. It was also difficult to determine whether industry funding had a true impact on reporting quality as many studies did not report their funding source in the publication but may have had connections to pharmaceutical companies. All CONSORT items were also weighed equally with regards to importance through the use of a median score, although some items may be considered as more important than others. However, as there is no available validated tool that assigns a score based on weighted items, the use of a median percentage was deemed most appropriate.

## CONCLUSIONS

Among RCTs evaluating interventions for aesthetic rhinoplasty, adherence to the CONSORT Harms tool was poor overall, highlighting a lack of transparency surrounding the potential complications of interventions investigated. This poor reporting quality makes it difficult for readers to make informed decisions about optimal techniques for aesthetic rhinoplasty. Future trials should follow the CONSORT Harms checklist to ensure that harms are clearly reported and to avoid solely focusing on the aesthetic benefits of treatment. The development of a consensus on harms to assess in future trials on aesthetic rhinoplasty should be considered.

## Supplemental Material

This article contains supplemental material located online at www.asjopenforum.com.

## Supplementary Material

ojae057_Supplementary_Data
