# Supplementary material for: Assessment of Harms Reporting Quality in Randomized Controlled Trials of Aesthetic Rhinoplasty: A Systematic Review
Source: Aesthet Surg J Open Forum. 2024 Jul 22;6:ojae057. doi: 10.1093/asjof/ojae057 (PMC11304942; doi:10.1093/asjof/ojae057)
Supplement: ojae057_Supplementary_Data [file ojae057_supplementary_data.zip › Appendix.docx]

**Appendix A: PRISMA 2020 Checklist**

| **Section and Topic** | **Item #** | **Checklist item** | **Location where item is reported** |
| --- | --- | --- | --- |
| **TITLE** | | |  |
| Title | 1 | Identify the report as a systematic review. | Title page |
| **ABSTRACT** | | |  |
| Abstract | 2 | See the PRISMA 2020 for Abstracts checklist. | 1 |
| **INTRODUCTION** | | |  |
| Rationale | 3 | Describe the rationale for the review in the context of existing knowledge. | 2 |
| Objectives | 4 | Provide an explicit statement of the objective(s) or question(s) the review addresses. | 2 |
| **METHODS** | | |  |
| Eligibility criteria | 5 | Specify the inclusion and exclusion criteria for the review and how studies were grouped for the syntheses. | 3 |
| Information sources | 6 | Specify all databases, registers, websites, organisations, reference lists and other sources searched or consulted to identify studies. Specify the date when each source was last searched or consulted. | 3 |
| Search strategy | 7 | Present the full search strategies for all databases, registers and websites, including any filters and limits used. | Appendix B |
| Selection process | 8 | Specify the methods used to decide whether a study met the inclusion criteria of the review, including how many reviewers screened each record and each report retrieved, whether they worked independently, and if applicable, details of automation tools used in the process. | 3 |
| Data collection process | 9 | Specify the methods used to collect data from reports, including how many reviewers collected data from each report, whether they worked independently, any processes for obtaining or confirming data from study investigators, and if applicable, details of automation tools used in the process. | 3 |
| Data items | 10a | List and define all outcomes for which data were sought. Specify whether all results that were compatible with each outcome domain in each study were sought (e.g. for all measures, time points, analyses), and if not, the methods used to decide which results to collect. | 3 |
|  | 10b | List and define all other variables for which data were sought (e.g. participant and intervention characteristics, funding sources). Describe any assumptions made about any missing or unclear information. | 3 |
| Study risk of bias assessment | 11 | Specify the methods used to assess risk of bias in the included studies, including details of the tool(s) used, how many reviewers assessed each study and whether they worked independently, and if applicable, details of automation tools used in the process. | NA |
| Effect measures | 12 | Specify for each outcome the effect measure(s) (e.g. risk ratio, mean difference) used in the synthesis or presentation of results. | NA |
| Synthesis methods | 13a | Describe the processes used to decide which studies were eligible for each synthesis (e.g. tabulating the study intervention characteristics and comparing against the planned groups for each synthesis (item #5)). | NA |
|  | 13b | Describe any methods required to prepare the data for presentation or synthesis, such as handling of missing summary statistics, or data conversions. | NA |
|  | 13c | Describe any methods used to tabulate or visually display results of individual studies and syntheses. | 3 |
|  | 13d | Describe any methods used to synthesize results and provide a rationale for the choice(s). If meta-analysis was performed, describe the model(s), method(s) to identify the presence and extent of statistical heterogeneity, and software package(s) used. | 4 |
|  | 13e | Describe any methods used to explore possible causes of heterogeneity among study results (e.g. subgroup analysis, meta-regression). | NA |
|  | 13f | Describe any sensitivity analyses conducted to assess robustness of the synthesized results. | NA |
| Reporting bias assessment | 14 | Describe any methods used to assess risk of bias due to missing results in a synthesis (arising from reporting biases). | 3 |
| Certainty assessment | 15 | Describe any methods used to assess certainty (or confidence) in the body of evidence for an outcome. | NA |
| **RESULTS** | | |  |
| Study selection | 16a | Describe the results of the search and selection process, from the number of records identified in the search to the number of studies included in the review, ideally using a flow diagram. | 4 |
|  | 16b | Cite studies that might appear to meet the inclusion criteria, but which were excluded, and explain why they were excluded. | NA |
| Study characteristics | 17 | Cite each included study and present its characteristics. | Appendix C |
| Risk of bias in studies | 18 | Present assessments of risk of bias for each included study. | NA |
| Results of individual studies | 19 | For all outcomes, present, for each study: (a) summary statistics for each group (where appropriate) and (b) an effect estimate and its precision (e.g. confidence/credible interval), ideally using structured tables or plots. | NA |
| Results of syntheses | 20a | For each synthesis, briefly summarise the characteristics and risk of bias among contributing studies. | Table 2 |
|  | 20b | Present results of all statistical syntheses conducted. If meta-analysis was done, present for each the summary estimate and its precision (e.g. confidence/credible interval) and measures of statistical heterogeneity. If comparing groups, describe the direction of the effect. | 5 |
|  | 20c | Present results of all investigations of possible causes of heterogeneity among study results. | NA |
|  | 20d | Present results of all sensitivity analyses conducted to assess the robustness of the synthesized results. | NA |
| Reporting biases | 21 | Present assessments of risk of bias due to missing results (arising from reporting biases) for each synthesis assessed. | Table 2 |
| Certainty of evidence | 22 | Present assessments of certainty (or confidence) in the body of evidence for each outcome assessed. | NA |
| **DISCUSSION** | | |  |
| Discussion | 23a | Provide a general interpretation of the results in the context of other evidence. | 5-7 |
|  | 23b | Discuss any limitations of the evidence included in the review. | 7-8 |
|  | 23c | Discuss any limitations of the review processes used. | 7-8 |
|  | 23d | Discuss implications of the results for practice, policy, and future research. | 8 |
| **OTHER INFORMATION** | | |  |
| Registration and protocol | 24a | Provide registration information for the review, including register name and registration number, or state that the review was not registered. | 2 |
|  | 24b | Indicate where the review protocol can be accessed, or state that a protocol was not prepared. | 2 |
|  | 24c | Describe and explain any amendments to information provided at registration or in the protocol. | NA |
| Support | 25 | Describe sources of financial or non-financial support for the review, and the role of the funders or sponsors in the review. | Title page |
| Competing interests | 26 | Declare any competing interests of review authors. | Title page |
| Availability of data, code and other materials | 27 | Report which of the following are publicly available and where they can be found: template data collection forms; data extracted from included studies; data used for all analyses; analytic code; any other materials used in the review. | Title page |

*From:*  Page MJ, McKenzie JE, Bossuyt PM, Boutron I, Hoffmann TC, Mulrow CD, et al. The PRISMA 2020 statement: an updated guideline for reporting systematic reviews. BMJ 2021;372:n71. doi: 10.1136/bmj.n7

**Appendix B: Search Strategy**

**Search Name: CENTRAL CONSORT HARMS**

**Date Run: 08/08/2023 10:38:38**

**Comment:**

**ID Search Hits**

**#1 MeSH descriptor: [Cosmetic Techniques] explode all trees 2409**

**Database:**
Embase <1974 to 2023 August 04>

| **#** | **Query** | **Results from 8 Aug 2023** |
| --- | --- | --- |
| 1 | exp esthetic surgery/ | 17,534 |
| 2 | randomized controlled trial/ | 776,623 |
| 3 | 1 and 2 | 428 |
| 4 | limit 3 to (english language and yr="2005 -Current") | 379 |

**Database:**
Ovid MEDLINE(R) and Epub Ahead of Print, In-Process, In-Data-Review & Other Non-Indexed Citations, Daily and Versions <1946 to August 04, 2023>

| **#** | **Query** | **Results from 8 Aug 2023** |
| --- | --- | --- |
| 1 | exp Esthetics/ | 16,529 |
| 2 | randomized controlled trial/ | 597,400 |
| 3 | 1 and 2 | 475 |
| 4 | limit 3 to (english language and yr="2005 -Current") | 401 |

**Appendix C: Characteristics of Individual Studies**

| Reference | Country | Blinding | Single- or multi-centre | Primary trial objective | Patient-important outcomes assessed | Sample size | Intervention group(s), including characteristics of rhinoplasty | Comparator group | Funding source |
| --- | --- | --- | --- | --- | --- | --- | --- | --- | --- |
| Akyigit et al., 2021 | Turkey | Single | Single | Efficacy | Nasal skin sensation | 184 | 1) Primary open rhinoplasty with lateral osteotomy 2) Revision rhinoplasty (open with lateral osteotomy) | No surgery | None |
| Alan et al., 2023 | Turkey | Non-blinded | Single | Efficacy | Nasal obstruction | 34 | Closed preservation rhinoplasty with lateral, radix, and transverse osteotomies | Open structural rhinoplasty with lateral osteotomies and bilateral medial oblique osteotomies | None |
| Amali, Sazgar, and Jafari, 2013 | Iran | Double | Single | Efficacy | Nasal obstruction | 52 | Open rhinoplasty with horizontal resection with cephalic hinged flap of the lateral crura of the lower lateral cartilage | Open rhinoplasty with cephalic trim of the lateral crura | None |
| Atighechi et al., 2018 | Iran | Non-blinded | Single | Efficacy | Nasal patency (swelling, obstruction, respiratory problems, difficulty during sleep and exercise) | 210 | 1) Open rhinoplasty with use of spreader graft 2) Open rhinoplasty with use of mattress suture | Open rhinoplasty without graft or mattress suture | NR |
| Aydoğdu and Bayram, 2020 | Turkey | Single | Single | Efficacy | Edema Ecchymosis Pain Obstruction and smelling function | 72 | Open rhinoplasty with lateral osteotomy using piezosurgery | Open rhinoplasty using conventional external lateral osteotomy | NR |
| Bai et al., 2020 | China | Double | Single | Efficacy | Post-operative mobility and appearance of nose Impact of procedure on social life | 200 | 1) Rhinoplasty through vestibular incision with the traditional carving method of L-shaped silicon graft 2) Rhinoplasty through vestibular incision with the traditional carving method of e-PTFE graft  3) Rhinoplasty through vestibular incision with double-V carving method of L-shaped silicon graft 4) Rhinoplasty through vestibular incision with double-V carving method of e-PTFE graft | No surgery | Government |
| Barone et al., 2020 | Italy | Non-blinded | Single | Efficacy | FACE-Q Rhinoplasty Module | 101 | 1) Open rhinoplasty with camouflage of the dorsum with diced cartilage 2) Open rhinoplasty with camouflage of the dorsum with lipofilling; 3) Open rhinoplasty with camouflage of the dorsum by a temporal fascia graft | Open rhinoplasty without camouflage of the dorsum | NR |
| Bertossi et al., 2021 | Italy | Non-blinded | Single | Efficacy | Patient satisfaction Major complications (tissue necrosis, infections) | 50 | Laser-assisted open rhinoplasty (laser used for resection of the crura, nasal hump, and osteotomies) | Classic open rhinoplasty | NR |
| Cabbarzade et al., 2021 | Turkey | Non-blinded | Single | Efficacy | Periorbital edema and ecchymosis | 40 | Open rhinoplasty with hump excision and lateral osteotomy and external splint used after surgery | Open rhinoplasty with hump excision and lateral osteotomy and surgical taping, without an external splint | None |
| Caglar et al., 2016 | Turkey | Single | Single | Efficacy | Periorbital edema and ecchymosis | 65 | Open septorhinoplasty with medial and lateral osteotomies following upper lateral cartilage modifications and tip plasty shortly before the end of the surgical operation | Open septorhinoplasty with medial and lateral osteotomies before upper lateral cartilate modifications and tip plasty | NR |
| Chan et al., 2019 | Canada | Single | Single | Efficacy | Periorbital edema and ecchymosis | 34 | Rhinoplasty with creation of subperiosteal tunnels prior to lateral osteotomies | Rhinoplasty with lateral and medial osteotomies without creation of subperiosteal tunnels | NR |
| Darzi et al., 2021 | Iran | Single | Single | Efficacy | Nasal function and patient satisfaction with cosmesis | 54 | Open rhinoplasty with medial and lateral osteotomies and lateral crural cut overlay | Open rhinoplasty with medial and lateral osteotomies and medial crural cut overlay | None |
| Dayan and Ashourian, 2016 | United States | Single | Single | Efficacy and harm | Rate of infection, rejection, extrusion of the graft, and tip projection | 30 | Closed rhinoplasty with caudal septal extension graft maneuver with use of polydioxanone absorbable plate | Closed rhinoplasty with caudal septal extension graft maneuver without use of polydioxanone absorbable plate | Industry |
| Dayan et al., 2018 | United States | Single | Single | Efficacy and harm | Edema Adverse events Ecchymoses Effect on daily activities Self-esteem  Aesthetic improvement | 10 | Closed rhinoplasty with lateral osteotomies with use of ARTISS human fibrin sealant | Closed rhinoplasty with lateral osteotomies without use of ARTISS human fibrin sealant | Industry |
| Deggeller et al., 2018 | Switzerland | Non-blinded | Single | Efficacy | Patient satisfaction Investigator-rated deformities (crooked nose, nasal hump) | 43 | 1) Open or closed rhinoplasty with osteotomies and use of NNF device immediately following surgery 2) Open or closed rhinoplasty with osteotomies without use of NNF device immediately following surgery | Use of NNF device without previous surgery | NR |
| Demirbilek and Evren, 2019 | Turkey | Single | Single | Efficacy and harm | Edema Inflammation Necrosis | 30 | Open rhinoplasty with excision and osteotomies performed via piezoelectric surgery | Open rhinoplasty with median and lateral osteotomies performed via osteotomes | NR |
| Erdogan et al., 2021 | Turkey | Single | Single | Efficacy | Periorbital edema and ecchymosis | 40 | Open rhinoplasty with lateral and internal median oblique osteotomies, followed by application of a 3D custom external nasal splint | Open rhinoplasty with lateral and internal median oblique osteotomies, followed by application of a thermoplastic nasal splint | Government |
| Erol et al., 2020 | Turkey | Single | Single | Efficacy and harm | Post-operative infection Wound healing Scar appearance Patient satisfaction Discomfort Pain | 64 | Open rhinoplasty with absorbable sutures | Open rhinoplasty with non-absorbable sutures | Private university |
| Fallahi et al., 2019 | Iran | Double | Single | Efficacy | Post-operative pain Edema Ecchymoses | 20 | Open rhinoplasty with internal lateral osteotomy using a piezosurgery device | Open rhinoplasty with conventional intranasal lateral osteotomy using an osteotome | NR |
| Ferreira et al., 2021 | Portugal | Non-blinded | Single | Efficacy | Nasal appearance Body image Nasal patency Quality of life | 250 | Open or closed rhinoplasty using component dorsal reduction (lateral osteotomies used as needed) | Open or closed rhinoplasty using lateral osteotomies with spare roof technique | None |
| Gentile and Cervelli, 2022 | Italy | Non-blinded | Single | Efficacy | Patient satisfaction Visual appearance Adverse effects | 35 | Open or closed rhinoplasty with lateral crural steal alar cartilage-modifying technique | Open or closed rhinoplasty with tongue in groove suture technique | NR |
| Gentile, 2023 | Italy | Non-blinded | Single | Efficacy | Patient satisfaction Visual appearance Adverse effects | 32 | Surgical fat grafting in subcutaneous tissue | Non-surgical hyaluronic acid injections | None |
| Ghavimi et al., 2018 | Iran | Double | Single | Efficacy | Post-operative edema and ecchymosis | 66 | Open rhinopladty with lateral nasal osteotomies via osteotomes | Open rhinoplasty with lateral osteotomies via piezosurgery | None |
| Ghazipour et al., 2014 | Iran | Double | Single | Efficacy | Post-operative edema and ecchymosis | 50 | Closed rhinoplasty with buccal sulcus lateral osteotomy | Closed rhinoplasty with intranasal lateral osteotomy | NR |
| Gode et al., 2018 | Turkey | Non-blinded | Single | Efficacy | Nasal obstruction Appearance Post-operative complications (irregular contour, asymmetry, persistent nasal obstruction, hematoma, septal perforation, infection) | 40 | In situ open septorhinoplasty with spreader grafts (ISS) with osteotomies as needed | Open septorhinoplasty with extracorporeal subtotal septal reconstruction and osteotomies as needed | NR |
| Gu et al., 2018 | China | Non-blinded | Single | Harm | Patient satisfaction, post-operative complications (infection, soft-tissue reaction, extrusion, irregularity) | 129 | Open rhinoplasty with expanded polytetrafluoroethylene alloplast for tip and dorsum augmentation | Open rhinoplasty with expanded polytetrafluoroethylene alloplast for tip and dorsum augmentation with conchal cartilage as a shield graft | None |
| Hashemi et al., 2021 | Iran | Double | Single | Efficacy | Satisfaction with shape of nasal tip and breathing | 45 | Open rhinoplasty with osteotomies and lateral crural overlay technique to correct cephalic malposition | Open rhinoplasty with osteotomies and lateral crural transposition technique to correct cephalic malposition | NR |
| Hashemi et al., 2005 | Iran | Single | Single | Efficacy | Post-operative edema and ecchymosis | 30 | Closed or open rhinoplasty with external lateral osteotomy | Closed or open rhinoplasty with internal lateral osteotomy | NR |
| Hernot et al., 2022 | India | Non-blinded | Single | Efficacy | Nasal obstruction, aesthetic appearance | 40 | Open rhinoplasty with bilateral triple osteotomies and septoplasty | Open rhinoplasty with bilateral triple ostetomies with spreader grafts and septoplasty | NR |
| Ince and Dadaci, 2017 | Turkey | Non-blinded | Single | Efficacy | Aesthetic appearance and function Patient satisfaction Complications | 166 | Open rhinoplasty with lateral osteotomies and wedge resection of bony nasal pyramid from base of nasal bone | Open rhinoplasty with lateral osteotomies and oblique hump resection | None |
| Jonas et al., 2022 | United States | Single | Single | Efficacy | Scar appearance Scar symptoms and satisfaction Complications | 41 | Open rhinoplasty with absorbable suture for columellar incision closure | Open rhinoplasty with permanent nonabsorbable suture for columellar incision closure | None |
| Kara, Kara, and Topuz, 2005 | Turkey | Single | Single | Efficacy | Post-operative periorbital edema Ecchymosis Subconjunctival ecchymosis Complications | 18 | Open or closed rhinoplasty with lateral osteotomies with subperiosteal tunnel | Open or closed rhinoplasty with lateral osteotomies without subperiosteal tunnel | None |
| Sowerby et al., 2019 | Canada | Double | Single | Efficacy | Post-operative edema and ecchymosis | 16 | Open septorhinoplasty with lateral osteotomies and pressure applied to the side of the nose | Open septorhinoplasty with lateral osteotomies without pressure applied to the side of the nose | None |
| Koc, Koc and Erbek, 2017 | Turkey | Double | Single | Efficacy | Post-operative edema, ecchymosis, pain, and patient satisfaction | 65 | Open rhinoplasty with lateral osteotomies using piezosurgery | Open rhinoplasty with lateral osteotomies using conventional osteotomy | NR |
| Koçak et al., 2017 | Turkey | Double | Single | Efficacy | Post-operative edema, ecchymosis and pain | 49 | Open rhinoplasty with lateral osteotomies using piezosurgery (some patients recieved transverse osteotomies in addition) | Open rhinoplasty with lateral osteotomies using conventional osteotomy (some patients recieved transverse osteotomies in addition) | None |
| Mamanov et al., 2017 | Turkey | Double | Single | Efficacy | Nasal obstruction | 30 | Open septorhinoplasty with lateral osteotomies with spreader grafts | Open septorhinoplasty with lateral osteotomies and no spreader grafts | NR |
| Martino et al., 2021 | Italy | Double | Single | Efficacy | Aesthetics, post-operative infection | 142 | Open rhinoplasty with tip reconstruction and N-butyl-cyanoacrylate + Metacryloxysulfolane Adhesive glue and traditional suture to fix graft | Open rhinoplasty with tip reconstruction and only suture to fix graft | None |
| Nemati et al., 2013 | Iran | Single | Single | Efficacy | Post-operative complications | 80 | Closed rhinoplasty with supratip skin defatting | Closed rhinoplasty without defatting | University |
| Prado et al., 2006 | Chile | Single | Single | Efficacy | Patient satisfaction, edema, ecchymosis, pain, recovery period, complications | 22 | Open rhinoplasty with percutaneous osteotomies and sealed aerosolized glue | Open rhinoplasty with percutaneous osteotomies and no glue | NR |
| Pryor et al., 2008 | United States | Single | Single | Efficacy | Pain, edema, ecchymosis, rate of healing | 10 | Rhinoplasty with bilateral lateral osteotomies and fibrin sealant | Rhinoplasty with bilateral lateral osteotomies and no fibrin sealant | None |
| Sadeghi et al., 2009 | Iran | Double | Single | Efficacy | Nasal tip projection Patient satisfaction | 96 | Closed rhinoplasty using columellar strut technique- open rhinoplasty was used in severe cases | Closed rhinoplasty without columellar strut technique- open rhinoplasty was used in severe cases | University |
| Saedi et al., 2017 | Iran | Single | Single | Efficacy | Dorsal edema Complications | 90 | 1) Open rhinoplasty with lateral osteotomy and triamcinolone injection (16mg/mL) 2) Open rhinoplasty with lateral osteotomy and triamcinolone injection (8mg/mL) | Open rhinoplasty with lateral osteotomy and no injection of triamcinolone | None |
| Saedi et al., 2016 | Iran | Single | Single | Efficacy | Scar aesthetics | 58 | Open rhinoplasty with inverted V incision | Open rhinoplasty with stair-step incision | None |
| Sahin et al., 2015 | Turkey | Non-blinded | Single | Efficacy | Nasal obstruction | 21 | Open rhinoplasty with conchal cartilage autologous graft using back-to-back technique | Open rhinoplasty with conchal cartilage autologous graft using face-to-face technique | NR |
| Sakallioğlu et al., 2015 | Turkey | Non-blinded | Single | Efficacy | Periorbital edema and ecchymosis | 50 | Open septorhinoplasty with lateral osteotomies | Closed septorhinoplasty with lateral osteotomies | NR |
| Salari & Totonchi, 2011 | Iran | Single | Single | Efficacy | Patient satisfaction | 100 | Open tip rhinoplasty using "Goldman Tip" technique (some patients had closed rhinoplasty) | Open tip rhinoplasty using suture techniques of the lower lateral cartilage (some had closed rhinoplasty) | NR |
| Santos et al., 2021 | Portugal | Non-blinded | Single | Efficacy | Aesthetics | 200 | Open or closed rhinoplasty using spare roof technique with either diced cartilage or shaved cartilage | Open or closed rhinoplasty using component dorsal reduction technique with either diced cartilage or shaved cartilage | None |
| Şirinoğlu, 2017 | Turkey | Non-blinded | Single | Efficacy | Tip rotation and projection | 44 | Open rhinoplasty with lateral osteotomies, sutures, and columnar strut graft | Open rhinoplasty with lateral osteotomies and suture | NR |
| Taş, 2020 | Turkey | Single | Single | Efficacy | Pain Edema Ecchymosis Cast comfort | 60 | 1) Closed rhinoplasty with lateral and medial osteotomies and vibration treatment and nasal cast with elastic bandage 2) Closed rhinoplasty with lateral and medial osteotomies and thermoplastic cast with elastic head bandage | Closed rhinoplasty with lateral and medial osteotomies and normal cast | None |
| Taşkın et al., 2017 | Turkey | Double | Single | Efficacy | Edema and ecchymosis | 90 | Open rhinoplasty with lateral and median-oblique osteotomies using piezosurgery | Open rhinoplasty with lateral and median-oblique osteotomies using conventional osteotome | None |
| Tirelli et al., 2015 | Italy | Single | Single | Efficacy | Pain Edema Ecchymosis Scarring Epistaxis | 22 | Open rhinoplasty with medial nasal and lateral osteotomies using piezosurgery | Open rhinoplasty with medial nasal and lateral osteotomies using traditional osteotome | NR |
| Toutounchi et al., 2015 | Iran | Single | Single | Efficacy | Patient satisfaction Nasal tip rotation and projection | 80 | Open rhinoplasty with cutting of depressor septi nasi muscle | Open rhinoplasty without cutting of depressor septi nasi muscle | None |
| Tulaci et al., 2020 | Turkey | Single | Single | Efficacy | Edema Ecchymosis Satisfaction Pre- and post-operative anxiety | 64 | Open septorhinoplasty with lateral and transverse osteotomy and infraorbital taping | Open septorhinoplasty with lateral and transverse osteotomy and no infraorbital taping | NR |
| Varedi & Bohluli, 2015 | Iran | Single | Single | Efficacy | Patient satisfaction | 40 | Open rhinoplasty with external percutaneous perforating osteotomies and external splint extended over the osteotomy line | Open rhinoplasty with external percutaneous perforating osteotomies and external splint trimmed above osteotomy line | NR |
| Wang et al., 2022 | China | Single | Multi-centre | Efficacy | Aesthetic improvement | 132 | Non-surgical rhinoplasty using maximum of 1mL Restylane Lyft | No treatment | Industry |
| Yaberi et al., 2018 | Iran | Single | Single | Efficacy | Tip rotation and projection Patient satisfaction Nasal obstruction | 80 | Open rhinoplasty using columellar strut technique | Open rhinoplasty using tongue in groove technique | None |
| Zaher et al., 2022 | Egypt | Single | Single | Efficacy | Edema and ecchymosis | 40 | Open septorhinoplasty with lateral osteotomy using percutaneous perforating method | Open septorhinoplasty with lateral osteotomy using open sky access method | None |
| Zorlu et al., 2023 | Turkey | Non-blinded | Single | Harm | Auditory function (pure tone audiometry, distortion product otoacoustic emission, tympanometry) | 40 | Open septorhinoplasty with medial and lateral osteotomies and use of burrs for hump excision and osteoplasty for nasal deformity | Turbinate surgery and septoplasty without use of burrs | NR |
| *Abbreviations.* ePTFE = expanded polytetrafluoroethylene; NNF = Nasella Nose Former; NR = not reported | | | | | | | | | |

**Appendix D: Reference List for Included Studies**

[1. Alan M, Kahraman M, Yuksel F, Yucel A. Comparison of Dorsal Preservation and Dorsal Reduction Rhinoplasty: analysis of Nasal Patency and Aesthetic Outcomes by Rhinomanometry, NOSE and SCHNOS Scales. 2023;47(2):728‐734.](https://www.zotero.org/google-docs/?4kggJh)

[2. Akyigit A, Keleş E, Yıldırım Y, Karlıdağ T, Eroglu O, Kaygusuz İ, et al. Comparison of Changes in Nasal Skin Sensation After Primary and Revision Rhinoplasty Procedures Using Semmes-Weinstein Monofilament Testing. 2021;41(10):NP1295‐NP1300.](https://www.zotero.org/google-docs/?4kggJh)

[3. Amali A, Sazgar A, Jafari M. Assessment of Nasal Function After Tip Surgery With a Cephalic Hinged Flap of the Lateral Crura: a Randomized Clinical Trial. 2014;34(5):687‐695.](https://www.zotero.org/google-docs/?4kggJh)

[4. Atighechi S, Sarafraz Z, Baradaranfar M, Dadgarnia M, Zand V, Meybodian M, et al. The Effect of Spreader Graft and Mattress Suture Technique on Rhinoplasty in Patients With Nasal Hump Smaller Than 3 mm. 2018;29(8):2110‐2113.](https://www.zotero.org/google-docs/?4kggJh)

[5. Aydoğdu I, Bayram A. Comparison of Early and Long-Term Effects of Piezosurgery With Conventional Techniques for Osteotomies in Rhinoplasty. 2020;31(6):1539‐1543.](https://www.zotero.org/google-docs/?4kggJh)

[6. Bai S, Li D, Xu L, Duan H, Yuan J, Wei M. A Novel Method to Enhance Dynamic Rhinoplasty Outcomes: double “V” Carving for Alloplastic Grafts. 2020;99(4):262‐267.](https://www.zotero.org/google-docs/?4kggJh)

[7. Barone M, Cogliandro A, Salzillo R, Ciarrocchi S, Panasiti V, Coppola R, et al. The Role of Skin Thickness in the Choice of a Rhinoplasty Technique for Thin-Skinned Patients: analysis of Long-Term Results and Patient Satisfaction. 2020;44(5):1742‐1750.](https://www.zotero.org/google-docs/?4kggJh)

[8. Bertossi D, Marchetti A, Sbarbati A, Nocini P. Laser-Assisted Rhinoplasty: the Future Generation Rhinoplasty Technique to Preserve Anatomy? A Series of Patients Compared to Patients Undergoing Standard Open Rhinoplasty. 2021;147(2):364‐369.](https://www.zotero.org/google-docs/?4kggJh)

[9. Cabbarzade C, Yücel Ö, Sözen T, Ozgen B. External Splinting Is Not Mandatory After All Rhinoplasties: a Prospective Randomized Trial. 2021;86(4):376‐380.](https://www.zotero.org/google-docs/?4kggJh)

[10. Dayan S, Bacos J, Ho T, Gandhi N, Gutierrez-Borst S. A Pilot Study Evaluating the Efficacy and Safety of ARTISS Human Fibrin Sealant in External Rhinoplasty. 2018;42(2):590‐597.](https://www.zotero.org/google-docs/?4kggJh)

[11. Demirbilek N, Evren C. Is Piezoelectric Surgery Really Harmless to Soft Tissue? 2019;30(7):1966‐1969.](https://www.zotero.org/google-docs/?4kggJh)

[12. Erol O, Buyuklu F, Koycu A, Jafarov S, Gultekin G, Erbek S. Comparison of Rapid Absorbable Sutures with Nonabsorbable Sutures in Closing Transcolumellar Incision in Septorhinoplasty: short-term Outcomes. 2020;44(5):1759‐1765.](https://www.zotero.org/google-docs/?4kggJh)

[13. Gentile P, Cervelli V. Cartilage Remodeling in Nasal Tip Rhinoplasty Using “Lateral Crural Steal” and “Tongue in Groove” Strategies: a Randomized Controlled Trial. 2022;33(4):1099‐1103.](https://www.zotero.org/google-docs/?4kggJh)

[14. Gentile P. Rhinofiller: Fat Grafting (Surgical) Versus Hyaluronic Acid (Non-Surgical). Aesthetic Plast Surg. 2023;47(2):702–13.](https://www.zotero.org/google-docs/?4kggJh)

[15. Ghavimi M, Nezafati S, Yazdani J, Pourlak T, Amini M, Ghoreishizadeh A, et al. Comparison of edema and ecchymosis in rhinoplasty candidates after lateral nasal osteotomy using piezosurgery and external osteotomy. 2018;9(3):87‐93.](https://www.zotero.org/google-docs/?4kggJh)

[16. Gode S, Benzer M, Uslu M, Kaya I, Midilli R, Karci B. Outcome of in situ septoplasty and extracorporeal subtotal septal reconstruction in crooked noses: a randomized self-controlled study. 2018;42(1):234‐243.](https://www.zotero.org/google-docs/?4kggJh)

[17. Hernot S, Agrawal A, Kaintura M, Maithani T, Dogra R. A Comparative Study of Isolated Osteotomies Versus Osteotomies with Spreader Graft Placement to Correct Primary Deviated Nose. 2022;46(2):818‐829.](https://www.zotero.org/google-docs/?4kggJh)

[18. Ince B, Dadaci M. Base Nasal Bone Resection versus Oblique Nasal Bone Resection: a Comparative Study of the Outcomes for the Deviated Nose. 2017;139(1):29e‐37e.](https://www.zotero.org/google-docs/?4kggJh)

[19. Mamanov M, Batioglu-Karaaltin A, Inci E, Erdur Z. Effect of Spreader Graft on Nasal Functions in Septorhinoplasty Surgery. 2017;28(7):e618‐e621.](https://www.zotero.org/google-docs/?4kggJh)

[20. Prado A, Andrades P, Danilla S, Benitez S, Wisnia P. Use of aerosolized bovine-prepared fibrin glue for skin fixation after primary open rhinoplasty: a prospective randomized and controlled trial. 2006;30(5):568‐573.](https://www.zotero.org/google-docs/?4kggJh)

[21. Sahin M, Kasapoglu F, Demir U, Ozmen O, Coskun H, Basut O. Comparison of Clinical Results in Nasal Tip Augmentation Either Via Face to Face or Back to Back Technique With Autogenous Auricular Conchal Cartilage. 2015;26(7):2109‐2114.](https://www.zotero.org/google-docs/?4kggJh)

[22. Şirinoğlu H. The Effect of the Short and Floating Columellar Strut Graft and Septocolumellar Suture on Nasal Tip Projection and Rotation in Primary Open Approach Rhinoplasty. 2017;41(1):146‐152.](https://www.zotero.org/google-docs/?4kggJh)

[23. Tirelli G, Tofanelli M, Bullo F, Bianchi M, Robiony M. External osteotomy in rhinoplasty: piezosurgery vs osteotome. 2015;36(5):666‐671.](https://www.zotero.org/google-docs/?4kggJh)

[24. Toutounchi J, Biroon S, Banaem S, Toutounchi N, Nezami N, Salari B. Effect of the depressor septi nasi muscle modification on nasal tip rotation and projection. 2015;39(3):294‐299.](https://www.zotero.org/google-docs/?4kggJh)

[25. Tulaci K, Arslan E, Tulaci T, Tastan E, Yazici H. Evaluating the Effect of Infraorbital Region Taping Procedure on Patient Anxiety, Satisfaction, Edema, and Ecchymosis Level on Primary Septorhinoplasty. 2020;31(5):1322‐1326.](https://www.zotero.org/google-docs/?4kggJh)

[26. Wang X, Li B, Li Q. Restylane Lyft for Aesthetic Shaping of the Nasal Dorsum and Radix: a Randomized, No-Treatment Control, Multicenter Study. 2022;150(6):1225‐1235.](https://www.zotero.org/google-docs/?4kggJh)

[27. Zaher M, Elfeki B, Ismail K, Ismail T, Hegazy S. Early Postoperative Sequelae After Open Sky Access in Nasal Osteotomy: a Comparative Study. 2022;88(5):480‐484.](https://www.zotero.org/google-docs/?4kggJh)

[28. Zorlu M, Aysel A, Aydin E, Catli T. The Effects of Burr-Assisted Rhinoplasty on Hearing. 2023;34(3):881‐883.](https://www.zotero.org/google-docs/?4kggJh)

[29. Caglar E, Celebi S, Topak M, Develioglu N, Yalcin E, Kulekci M. How can periorbital oedema and ecchymose be reduced in rhinoplasty? 2016;273(9):2549‐2554.](https://www.zotero.org/google-docs/?4kggJh)

[30. Chan D, Roskies M, Jooya A, Samaha M. Postoperative Ecchymosis and Edema After Creation of Subperiosteal Tunnels in Rhinoplasty: a Randomized Clinical Trial. 2019;21(2):133‐136.](https://www.zotero.org/google-docs/?4kggJh)

[31. Darzi E, Sadeghi M, Amali A, Saedi B. Effect of lateral crural cut overlay and medial crural cut and overlay in creating and maintaining tip projection and rotation: a randomised single-blind trial. 2021;59(9):1067‐1073.](https://www.zotero.org/google-docs/?4kggJh)

[32. Dayan S, Ashourian N. Polydioxanone Absorbable Plate for Cartilaginous Grafting in Endonasal Rhinoplasty: a Randomized Clinical Trial. 2016;18(1):47‐53.](https://www.zotero.org/google-docs/?4kggJh)

[33. Deggeller M, Holzmann D, Soyka M. Prospective evaluation of a nonsurgical device for rhinoplasty. 2018;56(1):73‐81.](https://www.zotero.org/google-docs/?4kggJh)

[34. Erdogan M, Simsek T, Ugur L, Kazaz H, Seyhan S, Gok U. The Effect of 3D-Printed Custom External Nasal Splint on Edema and Ecchymosis After Rhinoplasty. 2021;79(7):1549.e1‐1549.e7.](https://www.zotero.org/google-docs/?4kggJh)

[35. Fallahi H, Keyhan S, Fattahi T, Mohiti A. Comparison of Piezosurgery and Conventional Osteotomy Post Rhinoplasty Morbidities: a Double-Blind Randomized Controlled Trial. 2019;77(5):1050‐1055.](https://www.zotero.org/google-docs/?4kggJh)

[36. Ferreira M, Santos M, E Carmo DO, Fertuzinhos A, E Sousa CA, Santos J, et al. Spare Roof Technique Versus Component Dorsal Hump Reduction: a Randomized Prospective Study in 250 Primary Rhinoplasties, Aesthetic and Functional Outcomes. 2021;41(3):288‐300.](https://www.zotero.org/google-docs/?4kggJh)

[37. Ghazipour A, Alani N, Ghavami Lahiji S, Akbari Dilmaghani N. Buccal sulcus versus intranasal approach for postoperative periorbital oedema and ecchymosis in lateral nasal osteotomy. 2014;42(7):1456‐1459.](https://www.zotero.org/google-docs/?4kggJh)

[38. Gu Y, Yu W, Jin Y, Chen H, Ma G, Chang S, et al. Safety and Efficacy of Cosmetic Augmentation of the Nasal Tip and Nasal Dorsum With Expanded Polytetrafluoroethylene: a Randomized Clinical Trial. 2018;20(4):277‐283.](https://www.zotero.org/google-docs/?4kggJh)

[39. Hashemi Jazi S, Ghazavi H, Amali A, Rahavi-Ezabadi S, Mavvaji M. Comparing the efficacy of lateral crural overlay versus transposition technique on cephalic malposition in septorhinoplasty. 2021;59(7):807‐813.](https://www.zotero.org/google-docs/?4kggJh)

[40. Hashemi M, Mokhtarinejad F, Omrani M. A comparison between external versus internal lateral osteotomy in rhinoplasty. 2005;10(1):10‐15.](https://www.zotero.org/google-docs/?4kggJh)

[41. Jonas R, Patel K, Rist T, Walker E, Oyer S. Patient and Observer Graded Rhinoplasty Scar Outcomes: a Randomized Controlled Trial of Fast Absorbing Versus Permanent Columellar Suture Closure. 2022;24(3):196‐201.](https://www.zotero.org/google-docs/?4kggJh)

[42. Kara C, Kara I, Topuz B. Does creating a subperiosteal tunnel influence the periorbital edema and ecchymosis in rhinoplasty? 2005;63(8):1088‐1090.](https://www.zotero.org/google-docs/?4kggJh)

[43. Kim M, Moore C, Chow W, Sowerby L. Intraoperative compression after lateral osteotomy to minimize postoperative periorbital ecchymosis and edema. 2019;9:S91.](https://www.zotero.org/google-docs/?4kggJh)

[44. Koc B, Koc E, Erbek S. Comparison of clinical outcomes using a Piezosurgery device vs. a conventional osteotome for lateral osteotomy in rhinoplasty. 2017;96(8):318‐326.](https://www.zotero.org/google-docs/?4kggJh)

[45. Koçak I, Doğan R, Gökler O. A comparison of piezosurgery with conventional techniques for internal osteotomy. 2017;274(6):2483‐2491.](https://www.zotero.org/google-docs/?4kggJh)

[46. Martino C, Salzano F, Martino D, Ralli M, De Vincentiis M, Maranzano M, et al. A Prospective Randomized Trial of N-butyl-cyanoacrylate + Metacryloxysulfolane Adhesive versus Suture Alone for Grafting in Rhinoplasty: 9 year Follow-up. 2021;130(5):483‐489.](https://www.zotero.org/google-docs/?4kggJh)

[47. Nemati S, Banan R, Alizadeh A, Leili E, Kerdari H. Ultrasonographic evaluation of long-term results of nasal tip defatting in rhinoplasty cases. 2013;123(9):2131‐2135.](https://www.zotero.org/google-docs/?4kggJh)

[48. Pryor S, Sykes J, Tollefson T. Efficacy of fibrin sealant (human) (Evicel) in rhinoplasty: a prospective, randomized, single-blind trial of the use of fibrin sealant in lateral osteotomy. 2008;10(5):339‐344.](https://www.zotero.org/google-docs/?4kggJh)

[49. Sadeghi M, Saedi B, Arvin Sazegar A, Amiri M. The role of columellar struts to gain and maintain tip projection and rotation: a randomized blinded trial. 2009;23(6):e47‐50.](https://www.zotero.org/google-docs/?4kggJh)

[50. Saedi B, Amali A, Arabpor M. Comparison of two concentrations of triamcinolone injection in the prevention of supratip edema after external rhinoplasty: a randomized trial. 2017;31(6):412‐415.](https://www.zotero.org/google-docs/?4kggJh)

[51. Saedi B, Amali A, Taibnama N, Most S. Comparison of the aesthetic results of two common incisions in external rhinoplasty: a randomized trial. 2016;30(4):310‐312.](https://www.zotero.org/google-docs/?4kggJh)

[52. Sakallioğlu Ö, Cingi C, Polat C, Soylu E, Akyigit A, Soken H. Open Versus Closed Septorhinoplasty Approaches for Postoperative Edema and Ecchymosis. 2015;26(4):1334‐1337.](https://www.zotero.org/google-docs/?4kggJh)

[53. Salari B, Totonchi J. Evaluation of the Goldman tip procedure and suture technique in tip rhinoplasty. 2011;64(4):467‐471.](https://www.zotero.org/google-docs/?4kggJh)

[54. Santos M, Ribeiro A, Almeida ESC, Santos J, Dourado N, Amarante J, et al. Shaved Cartilage Gel Versus Diced Cartilage on Final Dorsal Camouflage: prospective Study of 200 Patients. 2021;23(3):164‐171.](https://www.zotero.org/google-docs/?4kggJh)

[55. Taş S. The Effects of Vibration and Pressure Treatments in the Early Postoperative Period of Rhinoplasty. 2020;40(6):605‐616.](https://www.zotero.org/google-docs/?4kggJh)

[56. Taşkın Ü, Batmaz T, Erdil M, Aydın S, Yücebaş K. The comparison of edema and ecchymosis after piezoelectric and conventional osteotomy in rhinoplasty. 2017;274(2):861‐865.](https://www.zotero.org/google-docs/?4kggJh)

[57. Varedi P, Bohluli B. Do the Size and Extension of the External Nasal Splint Have an Effect on the Osteotomy, Brow Lines, and Long-Term Results of Rhinoplasty: a Prospective Randomized Controlled Trial of 2 Methods. 2015;73(9):1843.e1‐9.](https://www.zotero.org/google-docs/?4kggJh)

[58. Yaberi R, Amali A, Emami H, Saedi B. A comparison of the tongue-in-groove and columellar strut in creating and maintaining tip projection and rotation: a randomized single blind trial. 2018;41(3):293‐298.](https://www.zotero.org/google-docs/?4kggJh)
